# The relationship between circulating irisin levels and tissues AGE accumulation in type 2 diabetes patients

**DOI:** 10.1042/BSR20170213

**Published:** 2017-06-07

**Authors:** Zhu Li, Gang Wang, Yan-juan Zhu, Chen-guang Li, Yun-zhao Tang, Zhen-huan Jiang, Min Yang, Chang-Lin Ni, Li-ming Chen, Wen-yan Niu

**Affiliations:** 1 Key Laboratory of Hormones and Development (Ministry of Health), Tianjin Key Laboratory of Metabolic Diseases, Tianjin Metabolic Diseases Hospital and Tianjin Institute of Endocrinology, Tianjin Medical University, Tianjin 300070, China; 2 Department of Immunology, Key Laboratory of Immune Microenvironment and Disease of the Educational Ministry of China, Tianjin Medical University, Tianjin 300070, China; 3 Tianjin University of Traditional Chinese Medicine, Tianjin 300193 China

**Keywords:** advanced glycation end products, endothelial dysfunction, irisin

## Abstract

Advanced glycation end-products (AGEs), measured by skin autofluorescence (AF), are a factor in the development or worsening of many degenerative diseases, such as diabetes and atherosclerosis. Irisin levels have been associated with diabetes, endothelial dysfunction and atherosclerosis. The objective of the present study was to investigate whether circulating irisin levels are correlated with skin AF values in type 2 diabetes patients. A total of 362 Chinese type 2 diabetic patients and 100 age- and sex-matched healthy controls were recruited in the present study. Clinical characteristics, blood biochemistry and circulating irisin levels were measured. Skin AF was measured using an AGE reader. Circulating irisin levels were significantly lower, while skin AF values were increased in type 2 diabetes compared with controls (*P*<0.05 respectively). By dividing the distribution of skin AF values into tertiles, serum irisin levels gradually lowered with increasing skin AF values (*P*<0.05). After adjusting for covariates, multivariate stepwise regression analysis demonstrated that serum lower irisin levels were independently associated with skin AF (*P*=0.009). Circulating irisin levels were lower in type 2 diabetes patients compared with healthy controls. Lower levels of irisin are independently associated with elevated skin AF values, indicating that circulating irisin levels could be associated with AGEs accumulation, which is one of the reasons causing vascular complications in diabetic patients.

## Introduction

Advanced glycation end-products (AGEs) are generated via non-enzymatic reactions involving the ketone or aldehyde groups of sugars with the free amino groups of proteins, lipids or nucleic acids. AGEs might be important factors in the development or worsening of many degenerative diseases, such as diabetes, atherosclerosis, chronic renal failure and Alzheimer’s disease [1,2]. Furthermore, the exacerbated production of AGEs is one of the mechanisms of endothelial dysfunction, considered as an early marker of cardiovascular disease and type 2 diabetes [3]. AGEs promote cellular stress responses through the engagement of receptor of AGEs (RAGE). Meerwaldt et al. [4,5] developed and validated a non-invasive technique to quantify tissue AGEs through the measurement of skin autofluorescence (AF). Measuring tissue AGES based on the skin AF might be preferable over plasma measurement, as long-lived proteins accumulate in the tissues in which chronic complications develop [6]. Skin AF, associated with cardiovascular mortality and the degree of atherosclerosis, is elevated in end-stage renal disease, and correlates with carotid intima media thickness (IMT) [7,8].

Irisin, a novel myokine, is proteolytically processed from the product of the fibronectin type III domain containing 5 (*FNDC5*) gene prior to release into circulation. This protein is regulated through peroxisome proliferator-activated receptor γ (PPAR-γ) coactivator-1(PGC1)-α [9]. Irisin has also been regarded as an anti-inflammatory factor, correlated with diabetes and the severity of insulin resistance [10–12]. It has been reported that circulating irisin levels are positively associated with endothelium-dependent vasodilation (EDV) in newly diagnosed type 2 diabetic patients without clinical angiopathy, indicating that circulating lower irisin levels are tightly associated with endothelial dysfunction and could be a marker for early stage atherosclerosis in type 2 diabetes [13]. Hou et al. [14] reported that circulating lower irisin was independently associated with endothelial dysfunction. Recently, Wang et al. reported that lower irisin levels were associated with urine albumin and flow-mediated arterial dilation. The present study also indicated that lower irisin is associated with endothelial dysfunction and atherosclerosis [15].

In summary, irisin is considered a protective factor for endothelial dysfunction and atherosclerosis, although the reasons for these considerations remain unclear. The exacerbated production of AGEs is one of the mechanisms underlying endothelial dysfunction. In type 2 diabetes, endothelial dysfunction is an important factor in the development of type 2 diabetic vascular complications. We hypothesized that circulating irisin levels are reduced in diabetes patients and this could partly increase the production of AGEs, leading to the development of endothelial dysfunction. The relationship between circulating irisin levels and AGE accumulation in diabetic subjects is currently unknown. Therefore, in the present study, we investigated the relationship between circulating irisin levels and skin AF values in type 2 diabetic patients.

## Materials and methods

### Study subjects

From March 2013 to December 2014, a total of 362 Chinese type 2 diabetic patients (179 men and 183 women, aged 37–64 years, mean age 50.5 ± 8.3 years), were recruited from the Tianjin Metabolic Diseases Hospital (Tianjin, China). Diabetes was diagnosed according to the World Health Organization (WHO) criteria (1999). Subjects with fasting plasma glucose (FPG) levels  ≥ 7.0 mmol/l and 2 h post-load plasma glucose (2 h PG) levels  ≥ 11.1 mmol/l were diagnosed with diabetes mellitus. Patients with T1DM, defined by the abrupt onset of symptoms, such as polyuria, polydipsia or unexplained weight loss, ketonuria or a past history of diabetic ketoacidosis, lack of insulin reserve, based on the results of C-peptide assay (fasting: <0.3  pmol/ml; stimulated: <0.6  pmol/ml), and requirement of insulin from the time of diagnosis for the control of hyperglycaemia, were excluded. T2DM was defined as absence of ketosis, good β-cell reserve as shown by C-peptide assay (fasting: ≥0.6  pmol/ml; stimulated: ≥1.6  pmol/ml). The research protocol was approved through the local ethics committee and informed written consent was obtained from each subject. Information, such as age, sex, weight, height, body mass index (BMI), age of onset of diabetes mellitus, fasting glucose levels, physical exercise, other diabetes-associated anomalies and blood pressure, were collected from them on a predesigned questionnaire. During the same period, 100 healthy subjects (all from medical staff in our hospital) were selected as control subjects. Physical exercise was defined as subjects who walked at least 25–30 min a day and at least 3–4 days a week for at least half a year. Subjects who were obese (BMI >30 kg/m^2^) and those with malignant neoplasms, renal or liver diseases or endocrinological disease other than diabetes were excluded from the study. Also, no patient was taking any drugs, such as antihypertensive drugs (including b-blockers), diabetes medications, oestrogen supplements, thyroxine, diuretics, hypolipidaemic drugs. All subjects enrolled in the study gave informed consents.

### Biochemical analysis

The blood samples were obtained after an overnight fast. The concentrations of triglyceride (TG) (Vitros TRIG DTD, Johnson & Johnson, New Brunswick, NJ, U.S.A.), total cholesterol (TC), low-density lipoprotein cholesterol (LDL-C), high-density lipoprotein cholesterol (HDL-C) (Vitros CHOL DTD, Johnson & Johnson) and glucose (Vitros GLU DTD, Johnson & Johnson) were analysed using the Vitros Chemistry DT60 II System (Johnson & Johnson). The serum fasting insulin (Fins) concentration was measured through electrochemiluminescence immunoassay (Roche Elecsys Insulin Assay, Roche Diagnostics, Mannheim, Germany). HOMA-insulin resistance (HOMA-IR) was calculated based on fasting serum insulin (Flns, mU/ml) × fasting blood glucose (FPG, mmol/l)/22.5. Serum irisin concentrations were measured in duplicate using ELISA kits (Aviscera Biosciences, Santa Clara, CA), in accordance with the manufacturer’s instructions. The sensitivity of the assay was 0.2 ng/ml and the linear range of the standard was 5–500 ng/ml. Haemoglobin A1c (HbA1c) was measured through high-performance chromatography. C-reactive protein (CRP) was measured using a particle enhanced immunoturbidimetric assay.

### Measurement of skin AF

Skin AF was assessed using an AGE Reader (DiagnOptics Technologies BV, Groningen, the Netherlands). The AGE Reader is a non-invasive desktop device that uses the characteristic fluorescent properties of certain AGEs to estimate the levels of AGE accumulation in the skin. This method has been extensively validated and strongly correlates with individual AGE compounds measured in skin biopsy dermal tissue homogenates obtained from the same site as the skin AF measurement. The AGE Reader illuminates a skin surface of 4 cm^2^ through an excitation light source with a peak excitation of 370 nm. Emission light (fluorescence in the wavelength of 420–600 nm) and reflected excitation light (with a wavelength of 300–420 nm) from the skin is measured using a spectrometer. The skin AF is calculated as the ratio between the emission light and reflected excitation light, which is multiplied by 100 and expressed in arbitrary units. A series of three consecutive measurements were obtained per minute. The mean skin AF was calculated using these measurements, and this value was used in subsequent analyses. This method is observer-independent and has a 5% intrapatient coefficient of variation.

### Statistical analysis

The data are presented as the means ± S.D. or the means ± S.E.M. or median (interquartile range). Unpaired Student’s *t*test was used to examine differences in numerical variables between the two groups. The chi-square test was used to compare categorical and nominal variables. Datasets of more than two groups were compared using ANOVA or the Mann–Whitney U test with Tukey’s post hoc analysis. For the univariate analysis of the effects of each potential risk factor on AF, linear regression for continuous variables was performed. The independent association between AF and other independent variables was assessed through multiple stepwise regression analysis. Fins and HOMA-IR concentrations were log transformed prior to analysis. The differences were considered significant for a *P* value <0.05. All data were analysed using the Statistical Package for Social Sciences (SPSS 18.0 for windows, SPSS Inc., Chicago, IL, U.S.A.).

## Results

### Clinical characteristics and biochemical parameters of the subjects

The clinical characteristics and biochemical data for the control subjects and diabetic patients are summarized in Table 1. Compared with the control subjects, the values of BMI, HbA1C, TC, TG, LDL-C, CRP, Fins, HOMA-IR and skin AF were significantly increased (*P*<0.05), whereas the levels of irisin and HDL-C were significantly lowered (*P*<0.05) in type 2 diabetic patients. As shown, circulating levels of irisin and skin AF have the opposite performance in type 2 diabetic patients.

**Table 1:** Clinical and biochemical characteristics in control and type 2 diabetes subjects

Variables	Controls	Type 2 diabetic patients	*P*-values
Males/females	51/49	179/183	
Age (years)	50.8 ± 9.5	50.5 ± 8.3	0.221
BMI (kg/m^2^)	23.9 ± 3.4	25.7 ± 3.0	0.009
SBP (mmHg)	120.6 ± 8.1	122.3 ± 7.9	0.318
DBP (mmHg)	71.5 ± 5.1	72.6 ± 5.8	0.231
Physical exercise (*n*(%))	10 (25%)	37 (22.8%)	0.311
HbA1c (%)	5.3 ± 0.5	7.7 ± 1.2	<0.001
TC (mmol/l)	4.25 ± 0.53	5.78 ± 0.67	<0.001
TG (mmol/l)	1.38 ± 0.51	2.41 ± 0.78	<0.001
LDL-C (mmol/l)	2.01 ± 0.36	3.57 ± 0.61	<0.001
HDL-C (mmol/l)	1.23 ± 0.32	1.11±0.31	0.038
CRP (mg/l)	1.21 ± 0.61	2.93 ± 0.78	<0.001
Fins (mU/l)	9.3 ± 2.4	13.4 ± 4.7	<0.001
HOMA-IR	2.57 ± 1.36	4.47 ± 2.33	<0.001
Irisin (ng/ml)	24.35 ± 2.76	16.24 ± 5.16	<0.001
Skin AF (AU)	1.97 (1.77 – 2.18)	2.72 (1.74 – 3.71)	<0.001

SBP, systolic blood pressure; DBP, diastolic blood pressure.

### Circulating irisin concentrations and AF values in type 2 diabetes

**Figure 1:**
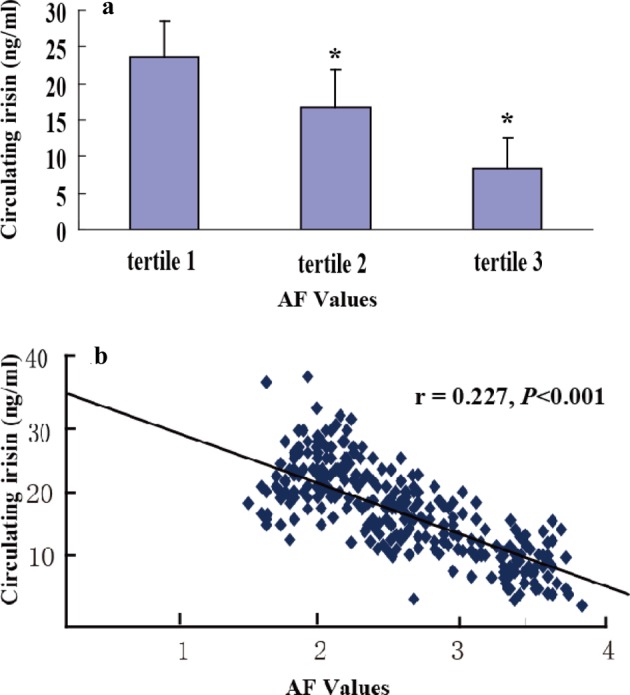
Correlations of circrlating irisn with skin AF values (**a**) Circulating irisin concentrations in different tertiles of skin AF values in type 2 diabetic patients. Data represent the mean ± S.E.M., *P<0.05 compared with tertile 1. (**b**) Correlation analysis to evaluate correlation of circulating irisin with AF values in diabetic patients.

The subjects were divided into three groups according to tertiles of AF values (median (interquartile range): 2.06 (1.74–2.38), 2.71 (2.41–3.01), 3.39 (3.08–3.71) respectively). There were no differences in age, gender, BMI, blood pressure, Fins (mU/l) and HOMA-IR among the three different tertile subgroups. Compared with the subjects in the lowest tertile of AF values, the patients in the highest tertile had significantly higher levels of HbA1c, TC, TG, LDL-C and CRP and lower levels of irisin and HDL-C (*P* for trend <0.05) (Table 2). Moreover, the irisin levels in tertiles 2 and 3 were significantly lower compared with those in tertile 1 after adjustment for age, gender and BMI (Figure 1).

**Table 2:** Clinical and biochemical characteristics in type 2 diabetic subjects according to the quartiles of skin AF values

Variables	Tertiles of skin AF values			*P-*value
	Tertile 1	Tertile 2	Tertile 3	
*n*	120	120	122	
Age (years)	50.2 ± 8.5	50.7 ± 7.9	50.5 ± 8.1	0.899
Sex (M/F)	54/66	59/61	66/56	0.334
Duration of diabetes (years)	10.3 ± 2.2	10.8 ± 2.4	10.5 ± 2.3	0.879
BMI (kg/m^2^)	24.9 ± 3.2	25.3 ± 3.1	26.2 ± 2.8	0.123
SBP (mmHg)	121.3 ± 7.8	123.1 ± 8.1	122.1 ± 8.2	0.135
DBP (mmHg)	72.4 ± 5.9	73.1 ± 5.4	72.3 ± 5.9	0.154
HbA1c (%)	7.1 ± 1.8	7.3 ± 1.5	8.7 ± 1.1	0.031
TC (mmol/l)	5.47 ± 0.67	5.79 ± 0.69	6.12 ± 0.71	<0.001
TG (mmol/l)	1.68 ± 0.75	2.43 ± 0.79	3.15 ± 0.74	<0.001
LDL-C (mmol/l)	2.97 ± 0.61	3.58 ± 0.68	4.16 ± 0.59	<0.001
HDL-C (mmol/l)	1.12 ± 0.31	1.13 ± 0.35	1.09 ± 0.34	0.059
CRP (mg/l)	1.76 ± 0.78	2.91 ± 0.77	4.12 ± 0.74	<0.001
Fins (mU/l)	13.7 ± 4.7	13.1 ± 4.9	13.5 ± 3.9	0.326
HOMA-IR	4.46 ± 2.33	4.49 ± 2.38	4.50 ± 2.29	0.599
Irisin (ng/ml)	23.59 ± 4.88	16.67 ± 5.23	8.46 ± 4.16	<0.001
Skin AF (AU)	2.06 (1.74 – 2.38)	2.71 (2.41 – 3.01)	3.39 (3.08 – 3.71)	<0.001

SBP, systolic blood pressure; DBP, diastolic blood pressure.

### Correlation and regression analysis

Univariate analysis showed a negative correlation between AF and circulating irisin (*r* =0.227, *P*<0.001), BMI (*r* = –0.21, *P*=0.033), systolic blood pressure (SBP) (*r* = –0.19, *P*=0.047), TC (*r* =0.217, *P*<0.001), LDL-C (*r* =0.23, *P*=0.004), TG (*r* =0.123,*P*=0.023), CRP (*r* = –0.31, *P*<0.001), HbA1c (*r* =0.251, *P*<0.001), log-HOMA-IR (*r* =0.256, *P*=0.021) and log-Fins (r = –0.260, *P*<0.001). To evaluate the independent association between AF and other independent variables, a multiple stepwise regression analysis was performed in diabetic patients. In this model, irisin, BMI, SBP, TC, LDL-C, TG, CRP, HbA1C, log-HOMA-IR and log-Fins were entered (forward) at the beginning of the procedure. Of these, irisin, HbA1C, LDL-C, TG and CRP were independently associated with the AF values (all *P*<0.05) (Table 3).

**Table 3:** Association of AF values with circulating irisin and biochemical parameters by multivariate stepwise regression analysis

Variables	β	S.E.M.	*P*-values
HbA1C (%)	0.986	0.241	<0.001
LDL-C (mmol/l)	0.199	0.218	0.044
TG (mmol/l)	0.561	0.226	0.012
CRP (mg/l)	0.986	0.241	<0.001
Irisin (ng/ml)	0.591	0.137	0.009

β, regression coefficient; adjustments for age, sex, BMI, SBP, diastolic blood pressure (DBP), smoking, positive family history and physical activity.

## Discussion

The results of the present study showed that circulating irisin levels were lower in type 2 diabetes patients compared with healthy controls. We demonstrated for the first time that lower levels of circulating irisin were independently associated with elevated skin AF, indicating increased AGE accumulation. To our knowledge, this is the first report to show the association of circulating irisin levels with AGE accumulation.

In the present study, we observed that AGE accumulation, based on the skin AF levels in type 2 diabetes patients, was higher than that in healthy controls (*P*<0.05). After dividing the distribution of skin AF values into tertiles, we observed that HbA1c levels gradually increased with increasing skin AF values (*P*<0.05), and multiple stepwise regression analysis showed that HbA1c levels were independently associated with AF values (*P*<0.05). AGEs are a complex and heterogeneous group of compounds implicated in diabetes-related complications. Reducing sugars, such as glucose, react non-enzymatically with amino groups in proteins, lipids and nucleic acids through a series of reactions forming Schiff bases and Amadori products to produce AGE. This process, is also known as the Maillard reaction. The amount of Amadori products is glucose concentration dependent [16]. An increasing number of studies have demonstrated that AGE accumulation leads to the aetiology of diabetic micro- and macrovascular complications [17]. Therefore, the glucose concentrations of blood and tissue contribute to the formation of AGEs and the development of diabetic vascular complications.

Irisin drives the conversion of white adipose tissues into brown fat-like adipose and mediates the beneficial effects of exercise on metabolism [9]. Circulating irisin was significantly reduced in type 2 diabetes patients compared with non-diabetic controls [12,13,18,19]. Here, we showed the same results. The levels of irisin were significantly lower (*P*<0.05) in type 2 diabetic patients than in control subjects.

Moreover, after dividing the distribution of skin AF values into tertiles, the serum irisin levels were gradually reduced with increasing skin AF values (*P*<0.05). Multivariate stepwise regression analysis demonstrated that serum reduced irisin levels were independently associated with elevated skin AF after adjusting for covariates (*P*=0.009). These results suggested that the lower circulating irisin levels might partially contribute to AGE accumulation in type 2 diabetes patients. The pathophysiological significance of the association between irisin and the accumulation of AGEs observed in the present study should be determined in future studies. However, there are some potential mechanisms that might account for this relationship in type 2 diabetes. Firstly, a previous study showed that irisin directly induced glucose and fatty acid uptake in human muscles via the AMPK pathway [20]. The overexpression of this myokine was sufficient to promote energy expenditure and alleviate insulin resistance in a diabetic animal model [9]. Considering the glucose concentrations of blood and tissue contributing to the formation of AGEs, the lower levels of irisin in type 2 diabetes will lead to the accumulation of AGEs. Secondly, Hou et al. [14] reported that circulating irisin was positively correlated with EDV and was independently associated with EDV, potentially reflecting endothelial function and the degree of arteriosclerosis. Similarly, Xiang et al. [13] discovered that circulating irisin levels were positively associated with flow-mediated dilation (FMD), which could detect the degree of EDV. A previous study reported that irisin plays a novel role in sustaining endothelial homoeostasis through the promotion of HUVEC proliferation via the ERK signalling pathway [21]. Therefore, in type 2 diabetes, lower irisin leads to partial endothelial dysfunction and promotes the occurrence of atherosclerosis. Reactive oxygen species (ROS) increased when atherosclerosis occurred. In addition, ROS generates the glycolysis intermediate products, as sources of increased AGE formation,and promote the formation of AGEs [22]. In conclusion, the lower levels of irisin in type 2 diabetes patients lead to AGE accumulation, which aggravates the degree of arteriosclerosis.

There are several limitations of the present study. For example, it would be better to include even larger sample sizes of type 2 diabetes patients. In addition, the correlation between plasma irisin and AGEs accumulation in the present clinical study provides strong evidence concerning the potential role of irisin in metabolic disorders, but does not address the cause–effect relationship in the pathology of angiopathy in type 2 diabetes.

In summary, the results of the present study provide the first clinical evidence supporting the negative correlation between serum irisin concentrations and AGE accumulation based on the levels of skin AF in type 2 diabetes patients. AGE accumulation is one of mechanisms of diabetic vascular complications. Illustration of this mechanism through *in vitro* and *in vivo* studies is needed to clarify whether irisin is a protective factor for the development of diabetic complications.

## Abbreviations

AGE: advanced glycation end-product
AF: skin autofluorescence
AMPK: AMP-activated protein kinase
BMI: body mass index
CRP: C-reactive protein
EDV: endothelium-dependent vasodilation
ERK: Extracellar signal regulated kinases
Fins: fasting insulin
FPG: fasting plasma glucose
HbA1c: haemoglobin A1c
HDL-C: high-density lipoprotein cholesterol
HOMA-IR: HOMA-insulin resistance
HOMA: The homeostasis Model Assessment
HUVEC: Human Umbilical vein endothelial cells
LDL-C: low-density lipoprotein cholesterol
ROS: reactive oxygen
SBP: systolic blood pressure
T1DM: type 1 diabetes mellitus
T2DM: type 2 diabetes mellitus
TC: total cholesterol
TG: triglyceride
